# Editorial: Advances on genomics and genetics of horticultural crops and their contribution to breeding efforts - volume II

**DOI:** 10.3389/fpls.2024.1385217

**Published:** 2024-02-27

**Authors:** Eleni Tani, Aliki Xanthopoulou, Christos Bazakos

**Affiliations:** ^1^ Laboratory of Plant Breeding and Biometry, Agricultural University of Athens, Athens, Greece; ^2^ Institute of Plant Breeding and Genetic Resources, ELGO-DIMITRA, Thessaloniki, Greece; ^3^ Joint Laboratory of Horticulture, ELGO-DIMITRA, Thessaloniki, Greece; ^4^ Department of Comparative Development and Genetics, Max Planck Institute for Plant Breeding Research, Cologne, Germany

**Keywords:** omics approaches, phylogenetics, breeding, genome editing, bioinformatics

## 1 Introduction

Horticultural crops harbor a multitude of beneficial attributes for human use. Research focus has shifted to horticultural crops with the goal of not only boosting production and quality but also resilience and sustainability (Lastochkina et al., 2022). The Research Topic of these manuscripts reports the recent advancements of -omic technologies, genome wide association studies as well as genome editing and their contribution in modern horticultural breeding.

Zhang et al., conducted a preparatory study of the mechanism of selenium accumulation and transportation in tea cultivars. Cai et al., used transcriptome analysis in order to study how fern gametophytes respond to different light conditions. Under the same framework, Scandola et al. through proteome and metabolome analysis, classified kale cultivars into two groups based on their amino acid and sugar content, as well as carbon and nitrogen metabolism, mRNA splicing, protein translation and light harvesting.

Hussain et al., conducted a genome-wide identification analysis of NRAMP gene family in *Kandelia obovate* and enhanced our understanding of the roles of *Kandelia obovata* NRAMPs in copper stress conditions. In the same aspect, Zhang et al., applied genome-wide identification analysis in order to explore PEBP gene family potential effect in flower development in pineapple. Another genome-wide identification study in *Ananas comosus* identified that GRF proteins are involved in the regulation of floral organ development and the response to gibberellin (Yi et al.).

Tripodi et al. described the creation of the first-ever SPET panel in lettuce and its use in population structure and genomic diversity analysis. By focusing on the cabbage CENH3 gene linked to haploid induction, Stajič and Kunej aimed to improve the chances for PEG-mediated protoplast transformation to increase editing efficiency using CRISPR/Cas9 in a non-model horticultural plant. The review paper by Rosa-Martinez et al. documented the research studies through both conventional and molecular breeding to increase the content of phenolic acids and flavonoids in three very important horticultural crops, namely tomato, eggplant and pepper. To provide insights into the evolutionary dynamics and phylogenetic relationships within Hordeum genus, Yuan et al. performed the sequencing, assembly and annotation of the chloroplast (cp) genomes of three wild perennial Hordeum species. In a similar manner, Lu and Li utilized a hybrid assembly strategy to assemble and annotate the mitogenome of *Syzygium samarangense* and enriched our understanding of Wax apple genetic features providing important information for its molecular breeding.

## 2 Molecular analyses through -omic approaches

Ferns, an ancient lineage of land plants, play a pivotal role in the alternation of generations (metagenesis) by nourishing the young sporophyte (Krieg and Chambers, 2022). One of the most important exogenous factors affecting plant growth is light. However, when a plant is exposed to excessive photo conditions, it can cause photostress (Takahashi and Badger, 2011). Cai et al., studied *Adiantum flabellulatum* gametophytes response to light and offered insights into the survival strategies of fern gametophytes in low-light environments. By employing transcriptome sequencing and gene expression analyses, the research has identified key genes and pathways involved in light sensitivity and photoprotection. These findings provide better understanding of the broader ecological dynamics of ferns and their evolutionary adaptations to shaded habitats.

Plants are important organic selenium sources for the human body. They uptake inorganic selenium and convert it into absorbable organic selenium (Ren et al., 2022). Tea plant (*Camellia sinensis* (L.) O. Kuntze) has a high ability to enrich Se (Ren et al., 2022). The manuscript of Zheng et al., provides a comprehensive analysis of selenium accumulation and transportation in different tea cultivars. Using transcriptome analysis, it unravels certain molecular mechanisms behind the varying abilities of tea plants to accumulate selenium. The study highlights key genes and pathways involved in this process, including those related to flavonoid biosynthesis and glutathione metabolism. The findings offer insights for agricultural practices and the health benefits of tea.

Kale contains a wealth of antioxidants, vitamins, minerals and fiber (Becerra-Moreno et al., 2014). The richness of its composition places it in the category of superfoods (Samec et al., 2019). Scandola et al., evaluated proteomics and metabolomics of diverse kale cultivars and marked a significant leap in the comprehension of kale’s nutritional dynamics through a systems-level analysis. By examining nine kale cultivars under controlled LED light conditions, the research unveils how the diel molecular activities influence kale’s growth, development, and nutritional content. With the use of plant phenotyping, proteomics, and metabolomics kale cultivars were categorized into two distinct groups based on their amino acid and sugar profiles. This study deepens our understanding of kale as a nutritious crop and paving the way for the adaptation of optimized cultivation strategies.

## 3 Genome-wide identification of plant gene families

Natural resistance-associated macrophage proteins (NRAMPs) are a class of metal transporters involved in metal uptake, transport and detoxification (Zhang et al., 2020; Tian et al., 2021). The study of Hussain et al., presents a detailed analysis of the NRAMP gene family in the mangrove species *Kandelia obovata*. It explores the genome-wide identification, structure, and expression of NRAMP genes, especially in response to varying levels of copper stress. This research enhances our knowledge of *Kandelia obovata* resilience mechanisms in harsh conditions, potentially aiding in the development of metal-tolerant crops.

The pineapple (*Ananas comosus* (L.) Merr.) is one of the most cultivated tropical fruits. Its flowering time exerts influence in yield and time of harvesting (Jin et al., 2021; Kim et al., 2022). PEBP gene family is involved in flower development as well as in the regulation of the flowering time (Jin et al., 2021; Kim et al., 2022). The manuscript of Zhang et al., delves into the PEBP gene family in pineapple (*Ananas comosus*). It identifies and categorizes 11 PEBP family members, exploring their phylogenetic relationships, chromosomal localization, and gene structure. Moreover, this research examines the expression patterns of these genes during pineapple flowering and flower development, highlighting the specific roles of certain genes, especially in response to ethylene treatment. These findings enrich our knowledge in understanding flowering mechanisms in pineapple.

Growth-regulating factor (GRF) proteins have been shown to impact plant growth and developmental processes (Kim et al., 2012; Lee et al., 2017; Zhang et al., 2021). Yi et al., focused on the GRF (Growth-Regulating Factor) gene family in pineapple. The study identified eight GRF transcription factor genes, categorized them into five subfamilies and explored their phylogenetic relationships, chromosomal locations, and expression profiles. Furthermore, their potential roles in floral organ development and response to gibberellin were revealed, contributing to penetration of pineapple’s growth, development, and stress responses. These results can be the springboard for further research on the regulatory mechanisms of GRF transcription factors during pineapple growth and development.

Single primer enrichment technology (SPET), provides the opportunity to carry out targeted genotyping of known polymorphisms and to find new random polymorphic loci and hundreds of unique SNPs (Scaglione et al., 2019). Tripodi et al. documented the development and use of the first SPET panel in lettuce and its utilization in population structure and genetic diversity analysis. A total of 81,531 SNPs were examined in a heterogeneous collection of 160 *Lactuca sativa* and *Lactuca serriola* accessions originating from different regions around the globe. The efficacy of SPET for GWAS was confirmed when associations were found in chromosomal areas that had previously been documented to host candidate genes for four major agricultural characteristics of lettuce. These results demonstrated the efficacy of SPET in enabling a more thorough characterization of lettuce collections.

## 4 Genome editing in cabbage

Clustered Regularly Interspaced Short Palindromic Repeats/CRISPR-associated systems (CRISPR/Cas9) are unquestionably becoming an essential tool in plant breeding (Zhu et al., 2020). The work presented by Stajič and Kunej targeted the cabbage CENH3 gene linked to haploid induction to optimize the conditions for PEG-mediated protoplast transformation to improve editing efficiency using CRISPR/Cas9 in a non-model horticultural plant. To effectively transfer the CRISPR/Cas9 vector into cabbage protoplasts, important parameters influencing transformation effectiveness, including PEG4000 concentration, incubation duration, and plasmid quantity, were assessed. These results could be applied not only to successful genome editing of cabbage and other brassicas, but also to studies involving transitory transformation techniques in protoplasts (i.e gene function analysis and subcellular localization).

## 5 Breeding for secondary metabolites content

The review paper by Rosa-Martinez et al. emphasizes on the importance of two of the largest classes of secondary metabolites, namely phenolic acids and flavonoids due their crucial functions regarding plant biology (i.e post-harvest life), plant resilience and health benefits (Cosme et al., 2020). More specifically, it provides detailed information on the progress throughout the years concerning breeding for higher accumulation of phenolic acids and flavonoids in some of the most popular vegetables worldwide, pepper, tomato, and eggplant, where a great variation in their accumulation profile has been observed (Rosa-Martınez et al., 2021). A wide range of genetic and genomic technologies has led to the discovery of structural enzymes, annotated genes and QTLs involved in the biosynthesis and accumulation of flavonoids and phenolic acids. The usefulness of combining phenotypic variability and molecular tools through conventional breeding and genetic engineering procedures has been documented with detailed information. Finally, possible negative effects of breeding for higher levels of phenolics are also mentioned, i.e research that has linked a higher phenolic content to a lower fruit organoleptic quality.

## 6 Non-nuclear genome assemblies and annotations

The assembly and annotation of non-nuclear genomes (mitochondrial and chloroplastic) are important for advancing our understanding of plant biology, phylogenetic evolution and genetic diversity while providing valuable information for the molecular breeding and genetic improvement (Kersten et al., 2016; Xue et al., 2019; Wang et al., 2022; Zhang et al., 2023b).

Wax apple (*Syzygium samarangense*) is a commercially important fruit belonging to one of the world’s most species-rich tree genera (Govaerts et al., 2008). Utilizing a hybrid assembly strategy, Lu and Li, successfully assembled and annotated a 530,242 bp circular mitogenome of *S. samarangense* cv Black Diamond (Zheng, 2011), revealing 61 unique genes and a plethora of genetic structures, including simple sequence, tandem and interspersed repeats. The in-depth exploration of the mitogenome’s evolutionary trajectory, marked by significant genomic reorganization and the loss of key protein-coding genes (Lu and Li, 2024). Furthermore, the identification of 591 RNA editing sites, particularly those leading to the gain or loss of start and stop codons, underscores the complexity of genetic regulation within this species. By conducting comprehensive analyses, including phylogeny, collinearity, and RNA editing validation, this study enriches our understanding of *S. samarangense* genetic features and provides important information for its molecular breeding (Lu and Li).

The sequencing, assembly and annotation of the chloroplast (cp) genomes of three wild perennial Hordeum species: *H. bogdanii*, *H. brevisubulatum*, and *H. violaceum*, revealed significant sequence variations and identified a series of genetic markers and hotspot regions, providing insights into the evolutionary dynamics and phylogenetic relationships within Hordeum (Yuan et al.). The comparative genomic study between wild and cultivated annual species, enriched the cp genome database and provided a valuable resource for the development of molecular markers for phylogenetic analysis and conservation efforts offering a comprehensive framework for exploring phylogenetic evolution and population genetics in the genus Hordeum (Yuan et al.).

## Author contributions

ET: Writing – original draft, Writing – review & editing. AX: Writing – original draft, Writing – review & editing. CB: Writing – original draft, Writing – review & editing.

## References

[B1] Becerra-Moreno A. Alanis-Garza P. A. Mora-Nieves J. L. Mora-Mora J. P. Jacobo-Velazquez D. A. (2014). Kale: an excellent source of vitamin c, pro-vitamin a, lutein and glucosinolates. Cyta-J Food 12, 298–303. doi: 10.1080/19476337.2013.850743

[B2] Cosme P. Rodrıguez A. B. Espino J. Garrido M. (2020). Plant phenolics:Bioavailability as a key determinant of their potential health-promoting applications. Antioxidants 9, 1263. doi: 10.3390/antiox9121263 33322700 PMC7764680

[B3] Govaerts R. H. A. Sobral M. Ashton P. Barrie F. R. Holst B. K. Landrum L.. (2008). World checklist of myrtaceae (London: Royal Botanic Gardens).

[B4] Jin S. Nasim Z. Susila H. Ahn J. H. (2021). Evolution and functional diversification of FLOWERING LOCUS T/TERMINAL FLOWER 1 family genes in plants. Semin. Cell Dev. Biol. 109, 20–30. doi: 10.1016/j.semcdb.2020.05.007 32507412

[B5] Kersten B. Faivre Rampant P. Mader M. Le Paslier M. C. Bounon R. Berard A.. (2016). Genome sequences of Populus tremula chloroplast and mitochondrion: Implications for holistic poplar breeding. PloS One 11, e0147209. doi: 10.1371/journal.pone.0147209 26800039 PMC4723046

[B6] Kim G. Rim Y. Cho H. Hyun T. K. (2022). Identification and functional characterization of FLOWERING LOCUS T in Platycodon grandiflorus. Plants (Basel) 11, 325. doi: 10.3390/plants11030325 35161306 PMC8840131

[B7] Kim J. -S. Mizoi J. Kidokoro S. Maruyama K. Nakajima J. Nakashima K.. (2012). Arabidopsis GROWTH-REGULATING FACTOR7 functions as a transcriptional repressor of abscisic acid– and osmotic stress–responsive genes, including DREB2A. Plant Cell 24, 3393–3405. doi: 10.1105/tpc.112.100933 22942381 PMC3462639

[B8] Krieg C. P. Chambers S. M. (2022). The ecology and physiology of fern gametophytes: A methodological synthesis. Appl. Plant Sci. 10, (2). doi: 10.1002/aps3.11464 PMC9039797 35495196

[B9] Lastochkina O. Aliniaeifard S. SeifiKalhor M. Bosacchi M. Maslennikova D. Lubyanova A. (2022). Novel approaches for sustainable horticultural crop production: advances and prospects. Horticulturae 8, 910. doi: 10.3390/horticulturae8100910

[B10] Lee S. -J. Lee B. H. Jung J. -H. Park S. K. Song J. T. Kim J. H. (2017). Growth-regulating factor and grf-interacting factor specify meristematic cells of gynoecia and anthers. Plant Physiol. 176, 717–729. doi: 10.1104/pp.17.00960 29114079 PMC5761776

[B11] Lu G Li Q. Complete mitochondrial genome of *Syzygium samarangense* reveals genomic recombination, gene transfer, and RNA editing events. Front. Plant Sci. (2024) 14, 1301164. doi: 10.3389/fpls.2023.1301164 38264024 PMC10803518

[B12] Ren H. Li X. Guo L. Wang L. Hao X. Zeng J. (2022). Integrative transcriptome and proteome analysis reveals the absorption and metabolism of selenium in tea plants (Camellia sinensis (L.) O. Kuntze). Front. Plant Sci. 13. doi: 10.3389/fpls.2022.848349 PMC8908381 35283867

[B13] Rosa-Martınez E. García-Martínez M. D. Adalid-Martínez A. M. Pereira-Dias, L. Casanova C. Soler E.. (2021). Fruit composition profile of pepper, tomato and eggplant varieties grown under uniform conditions. Food Res. Int. 147, 110531. doi: 10.1016/j.foodres.2021.110531 34399509

[B14] Samec D. Urlic B. Salopek-Sondi B. (2019). Kale (Brassica oleracea var. acephala) as a superfood: review of the scientific evidence behind the statement. Crit. Rev. Food Sci. 59, 2411–2422. doi: 10.1080/10408398.2018.1454400 29557674

[B15] Scaglione D. Pinosio S. Marroni F. Centa E. Di Fornasiero A. Magris G.. (2019). Single primer enrichment technology as a tool for massive genotyping: a benchmark on black poplar and maize. Ann. Bot. 124, 543–551. doi: 10.1093/aob/mcz054 30932149 PMC6821380

[B16] Takahashi S. Badger M. R. (2011). Photoprotection in plants: a new light on photosystem II damage. Trends Plant Sci. 16, 53–60. doi: 10.1016/j.tplants.2010.10.001 21050798

[B17] Tian W. He G. Qin L. Li D. Meng L. Huang Y.. (2021). Genome-wide analysis of the NRAMP gene family in potato (Solanum tuberosum): Identification, expression analysis and response to five heavy metals stress. Ecotoxicol. Environ. Saf. 208, 111661. doi: 10.1016/j.ecoenv.2020.111661 33396171

[B18] Wang N. Li C. Kuang L. Wu X. Xie K. Zhu A.. (2022). Pan-mitogenomics reveals the genetic basis of cytonuclear conflicts in citrus hybridization, domestication, and diversification. Proc. Natl. Acad. Sci. U. S. A. 119, e2206076119. doi: 10.1073/pnas.2206076119 36260744 PMC9618123

[B19] Xue S. Shi T. Luo W. Ni X Iqbal S Ni Z.. (2019). Comparative analysis of the complete chloroplast genome among *Prunus mume*, *P. armeniaca* and *P. salicina* . Hortic. Res. 6, 89. doi: 10.1038/s41438-019-0171-1 31666958 PMC6804877

[B20] Zhang B. Tong Y. Luo K. Zhai Z. Liu X. Shi Z.. (2021). Identification of GROWTH-REGULATING FACTOR transcription factors in lettuce (Lactuca sativa) genome and functional analysis of LsaGRF5 in leaf size regulation. BMC Plant Biol. 21, 1–13. doi: 10.1186/s12870-021-03261-6 34688264 PMC8539887

[B21] Zhang J. Zhang M. Song H. Zhao J. Shabala S. Tian S.. (2020). A novel plasma membrane-based NRAMP transporter contributes to Cd and Zn hyperaccumulation in Sedum alfredii Hance. Environ. Exp. Bot. 176, 104121. doi: 10.1016/j.envexpbot.2020.104121

[B22] Zhang S. Wang J. He W. Kan S. Liao X. Jordan D. R.. (2023b). Variation in mitogenome structural conformation in wild and cultivated lineages of sorghum corresponds with domestication history and plastome evolution. BMC Plant Biol. 23, 91. doi: 10.1186/s12870-023-04104-2 36782130 PMC9926791

[B23] Zheng D. (2011). Cultivation techniques for high quality and high yield of wax apple (Beijing: China: Agricultural Science and Technology Press).

[B24] Zhu H. Li C. Gao C. (2020). Applications of CRISPR-Cas in agriculture and plant biotechnology. Nat. Rev. Mol. Cell Biol. 21, 661–677. doi: 10.1038/s41580-020-00288-9 32973356

